# Linking patient satisfaction with nursing care: the case of care rationing - a correlational study

**DOI:** 10.1186/1472-6955-13-26

**Published:** 2014-09-03

**Authors:** Evridiki Papastavrou, Panayiota Andreou, Haritini Tsangari, Anastasios Merkouris

**Affiliations:** 1 Department of Nursing, School of Health Sciences, Cyprus University of Technology, Vragadinos street 15, Limassol, Cyprus; 2 Department of Economics and Finance, School of Business, University of Nicosia, Nicosia, Cyprus

**Keywords:** Nursing care, Rationing, Patient satisfaction, Professional environment

## Abstract

**Background:**

Implicit rationing of nursing care is the withholding of or failure to carry out all necessary nursing measures due to lack of resources. There is evidence supporting a link between rationing of nursing care, nurses’ perceptions of their professional environment, negative patient outcomes, and placing patient safety at risk. The aims of the study were:

a) To explore whether patient satisfaction is linked to nurse-reported rationing of nursing care and to nurses’ perceptions of their practice environment while adjusting for patient and nurse characteristics.

b) To identify the threshold score of rationing by comparing the level of patient satisfaction factors across rationing levels.

**Methods:**

A descriptive, correlational design was employed. Participants in this study included 352 patients and 318 nurses from ten medical and surgical units of five general hospitals. Three measurement instruments were used: the BERNCA scale for rationing of care, the RPPE scale to explore nurses’ perceptions of their work environment and the Patient Satisfaction scale to assess the level of patient satisfaction with nursing care. The statistical analysis included the use of Kendall’s correlation coefficient to explore a possible relationship between the variables and multiple regression analysis to assess the effects of implicit rationing of nursing care together with organizational characteristics on patient satisfaction.

**Results:**

The mean score of implicit rationing of nursing care was 0.83 (SD = 0.52, range = 0–3), the overall mean of RPPE was 2.76 (SD = 0.32, range = 1.28 – 3.69) and the two scales were significantly correlated (τ = −0.234, p < 0.001). The regression analysis showed that care rationing and work environment were related to patient satisfaction, even after controlling for nurse and patient characteristics. The results from the adjusted regression models showed that even at the lowest level of rationing (i.e. 0.5) patients indicated low satisfaction.

**Conclusions:**

The results support the relationships between organizational and environmental variables, care rationing and patient satisfaction. The identification of thresholds at which rationing starts to influence patient outcomes in a negative way may allow nurse managers to introduce interventions so as to keep rationing at a level at which patient safety is not jeopardized.

## Background

The current worldwide economic crisis has resulted in public spending reductions on health care in many countries. According to the Organisation for Economic Cooperation and Development’s (OECD) recent reports on public expenditure, many governments have tried to contain the growth in “one of the biggest ticket items in most countries”, namely hospital spending, by cutting wages, reducing hospital staff and beds, plus increasing co-payments for patients [1]. Although WHO recognizes nurses as frontline service providers [2], nursing is generally considered a “cost” rather than revenue in a hospital context, which makes nursing a constant target for cost reductions [3]. These cutbacks combined with the phenomenon of permanent shortages of nurses are making rationing of care an increasingly prominent feature in health care [4].

Implicit rationing of nursing care is the withholding of or failure to carry out all necessary nursing measures due to lack of nursing resources such as time, staffing or skill mix [5]. According to the conceptual framework of nursing care rationing developed by Schubert [6], such nursing measures include actions of surveillance, therapy, prevention, rehabilitation and support, and these actions are important in order to achieve desired outcomes for patients. Rationing of nursing care occurs at the patient-to-nurse interface, it is based on the nurses’ assessments and it is a product of clinical decision making and clinical judgment. The rationing process is influenced by a number of factors including patient and nurse variables, the characteristics of the work environment, organizational variables, the philosophy of care and it is linked to patient and nurse outcomes. The effect of the work environment on rationing is also stressed in the Missed Care Model [7]. The model argues that the factors underlying missed care are linked to the context of the care environment, they are external to nurses and create a need to decide what care will be provided.

These include the labour and material resources available to assist in patient care activities as well as relationship and communication factors that affect the ability of nurses to deliver care. However, although research into links between nursing care and patient outcomes is proliferating, there is a lack of accumulated knowledge regarding the association between patient satisfaction and rationing of nursing care within professional environmental constraints.

### Review of the literature

Research evidence supports that there is a link between rationing of nursing care and negative patient outcomes such as increased mortality [8], patient falls [9],10], low quality of care [11], pressure ulcers [4] and hospital acquired infections [4,9,12]. Kalisch et al. [7] places the issue within the patient safety movement suggesting that “acts of omission” are identified as one of the major types of errors not addressed in the literature.

Patient satisfaction is generally accepted as a crucial indicator of the quality and effectiveness of care [13] as well as an important part of value-based health care, and it appears to be particularly sensitive to rationing [4]. Theoretically, patient satisfaction is connected with nursing care, nurses, and the organisational environment [14]. Several environmental factors have been reported as hindering the nursing profession in its ability to achieve improved health outcomes through the provision of competent, culturally sensitive, evidence-based care [2,15]. These factors include poor working conditions, heavy workloads, lack of participation in decision making, and limited opportunities for career mobility. Consequently lack of resources, as well as professional, environmental and other restraints and limitations when combined with the invisibility of caring could lead to negative outcomes for patients, nurses and the health care system in general. Patient satisfaction due to care is a critical outcome because it influences adherence to treatment, health services utilization and general attitudes towards the health care system [16]. Apart from being an important indicator of quality nursing care [17], patient satisfaction has a reciprocal effect meaning it can be used to improve nursing care that will in turn increase satisfaction [13]. Several studies have demonstrated an association between nursing and patient satisfaction identifying nursing care as the only hospital service having a direct and strong relationship with overall patient satisfaction [18,19]. Other researchers identified that patient-perceived nurse caring is a major predictor of patient satisfaction [20,21]. A correlational study examining surgical patient satisfaction as an outcome of nurse caring in six European countries, reports that caring behaviors enacted by nurses determined a consistent proportion of patients’ satisfaction [16]. The authors found that 44.1% of satisfaction variance was explained by the nurse caring behaviours as perceived by the patients [16]. Similarly, patient satisfaction was examined as an outcome of individualised care providing further evidence that a specific dimension of care, that is “individualised” care, is related with patient satisfaction [22-24]. This association seems to be an international phenomenon as it is reported in cross-cultural studies claiming that a large proportion of the satisfaction variance is explained by the patients’ perceptions of the support and provision of individualised care [24].

A plethora of studies have also examined the relationship between nurses’ perceptions of their work environment and the quality of care patients receive showing that improved work environments were associated with increased ratings of care quality and patient satisfaction [11,25-29]. Some researchers have examined the specific contribution of nurses’ work environments to patient satisfaction indicating that patients’ reports of satisfaction are higher in hospital settings where nurses practice in better work environments [19,30]. On the other hand, an unstable environment is linked with negative patient outcomes including nursing tasks being delayed, patient falls, and medication errors in both medical and surgical departments [31,32].

Also there is evidence of a positive relationship between some aspects of the professional work environment such as leadership style, and higher patient satisfaction, lower patient mortality rates, medication errors, restraint use and hospital-acquired infections [33,34]. Similarly, a work environment that facilitates patient-centered care is considered to increase patient safety and nurse satisfaction. More specifically, Rathert and May [35] found that nurses whose work units were more patient-centered reported that medication errors occurred less frequently in their units, and felt more comfortable to report errors and near-misses than those in less patient-centered units. Aiming to investigate environmental dimensions predicting nursing care rationing in a cross-sectional multicenter study, Schubert et al. [6] found that better unit level staff resource adequacy and a more favorable hospital level safety climate were both consistently and significantly associated with lower rationing levels. Similarly a large study in twelve European countries, exploring nurses perceptions of their work environment and quality of care, showed that in most countries nurses were dissatisfied with their work and reported that essential nursing tasks were left undone [28].

Some studies have focused on rationing of nursing care and related concepts such as care omissions, delays [36] and care priority setting [37-39] and provide evidence of a relationship between nursing care rationing and patient negative outcomes. For example, Lucero et al. [40], Kalisch et al. [10] and Schubert et al. [6] showed that unmet care needs, missed nursing care and rationing of nursing care had significant effects on nurse-reported adverse events such as hospital acquired infections, patients receiving wrong medications or dosage errors, and more incidents of patient falls causing injury. The quality of care on the basis of nursing care deficiencies was also explored and indicated that a significant relationship existed between quality care and patient safety ratings, and also to rates of unfinished care [11,40,41].

Only two studies were found to provide evidence of interlinks among patient satisfaction, nursing care rationing and practice environment factors. In a sample of 1338 nurses and 779 patients, Schubert et al. [4] identified that patient satisfaction with care was adversely affected by even a low level of rationing, and was accompanied by a 57% decrease in the number of patients who reported being very satisfied with their care. In a later study another team [42] aiming to explore the relationship between patient safety climate and patient outcomes in Swiss acute care hospitals after adjusting for major organizational variables, found that higher levels of implicit rationing of nursing care resulted in 72% decrease in patient satisfaction.

However, both studies assessed satisfaction based on one question, a common practice in several studies. Nonetheless, a single item question does not allow exploring the different perspectives that comprise patient satisfaction related to nursing care.

### Purpose

The aims of the study were two-fold:

a) To explore whether patient satisfaction is related to nurse-reported rationing of nursing care and nurses’ perceptions of their care environment, and if so to what extent, while adjusting for patient and nurse characteristics.

b) To identify the threshold score of self-reported rationing by comparing the level of patient satisfaction factors across rationing levels

## Methods

### Design and sample

An explorative, descriptive, correlational design was employed. The study was carried out in the five acute care hospitals of the Cyprus Republic. These are public general hospitals directly under the administration of the Ministry of Health, as Cyprus is a small country with a highly centralized public administration system. Patients were recruited from all the surgical and medical departments of the above hospitals (ten units in total) via convenience sampling. The inclusion criteria were:

a) That the respondent had received care on an adult surgical or medical unit for at least two days.

b) They had just received their discharge.

c) They had the ability to give verbal consent for participation

d) They were able to answer the questionnaire independently.

Three hundred and fifty two (352) patients agreed to participate in the study. Nurses were recruited from the corresponding departments via convenience sampling. To be included in the study, nurses were required to be:

a) Registered according to national legislation, which is in line with the EU Directives,

b) Actively involved in direct patient care.

c) Willing to participate in the study.

Power analysis indicated that the minimum number of participants to get a power of 99% (α = 0.05) was 318 nurses.

### Research instruments

The patients completed the Patient Satisfaction Scale [43]. The nurses completed two research instruments together with a demographic data sheet: the Basel Extent of Rationing of Nursing Care (BERNCA) [5] and the Revised Professional Practice Environment (RPPE) [44,45].

#### The Patient Satisfaction scale

This was developed and validated in the Greek language [43,46] and adjusted for the Cypriot Greek speaking population by a panel of experts [47]. The scale consists of two main factors: factor A (direct nursing care) and factor B (indirect nursing care) and includes 29 questions in total. Factor A was further split into 3 subscales (A1: technical care - 9 items; A2: information - 4 items; A3: interpersonal relations – 7 items). All the items were measured on a 5-point Likert scale from 1 = completely dissatisfied up to 5 = completely satisfied. The satisfaction score of each factor and subscale is obtained by the average sum of the items.

#### The BERNCA scale

This was developed within a European context [5] assessing implicit rationing addressing areas such as activities of daily living, care and support, rehabilitation, surveillance and security, and documentation. The scale includes 20 negatively-phrased questions on a list of tasks related to the above areas, and nurses need to indicate the extend they are able to perform these in the past seven days. Responses are marked on a four-point Likert type scale (never, rarely, sometimes, or often, 0–3 respectively) and rationing score is obtained from the average sum of all items (mean score range: 0–3). The construct validity was confirmed with exploratory factor analysis and the results indicated a one factor solution which confirmed the instruments’ one-dimensional internal structure [5].

#### The Revised Professional Practice Environment (RPPE) scale

The evaluation of the nurse environment characteristics was based on the RPPE scale, a 39-item validated instrument. The participants are asked to indicate their agreement on a four-point Likert type scale (with strongly disagree, disagree, agree, strongly agree as 1–4 respectively) to statements-descriptions of their working environment. The scale comprises 8 sub-scales on: Handling Disagreement & Conflict (9 items); Internal Work Motivation (8 items); Control over Practice (5 items); Leadership and Autonomy in Clinical Practice (5 items); Staff Relationships with physicians (2 items); Teamwork (4 items); Cultural Sensitivity (3 items); Communication about patients (3 items). The score of each subscale is based on its mean item scores.

Low scores on the BERNCA suggest low levels of rationing whereas low scores on the RPPE suggest perceptions of low levels on professional practice environment. Both instruments have been translated following the guidelines of MAPI for translating and validating health research instruments for cross-cultural use [48] and they were used in international studies [49,50].

The reliability of the instruments was measured with the Cronbach’s alpha coefficient, which for the BERNCA scale alpha was 0.91, for the RPPE was 0.89 and for the patient satisfaction scale it was found to be 0.93.

### Data collection

The data collection period lasted 9 months between 2010 and 2011 and the questionnaires were distributed by researchers not involved in any nursing care and appointed in each setting by the research team. All the questionnaires were anonymous and participation was voluntary. The nurses were approached during their shifts and were informed in writing about the purpose of the study, including its voluntary nature, and with a guarantee of their anonymity and the confidentiality of the data. They were invited to complete the questionnaires in their own time and return them in a sealed envelope, which they placed in a marked box on their ward. Return of the questionnaire was considered as informed consent. The identification of patients who fulfilled the inclusion criteria was done by the researchers jointly with the nurse in charge of the ward on a daily basis; the researcher then approached the patients informing them both orally and in writing about the study, and supplied them with the questionnaire to be completed anonymously on their day of their discharge. Completion and return of the questionnaire to the researcher was considered as informed consent.

### Ethical considerations

Approval to conduct the study was obtained from the National Bioethics Committee (ΕΕΒΚ ΕΠ 2010.01.21) after the submission of a detailed research proposal. Access to hospital facilities was granted by the Ministry of Health and the administrators of each participating hospital separately.

### Statistical analysis

The data analysis was performed at the nurse and patient individual level, departmental level (i.e. surgical and medical wards), unit level (i.e. surgical and medical wards at each of the five hospitals thus ten units in total) and hospital level using techniques appropriate for their levels of measurement and data distributions. More specifically, descriptive statistics such as percentages, means and standard deviations were used to describe the sample characteristics, patient satisfaction, rationing of nursing care and perception of professional practice environment. Moreover, Kendall’s correlation coefficient was used to explore a possible relationship between rationing of nursing care and professional practice environment. Finally, the effects of implicit rationing of nursing care and organizational characteristics (independent variables) on the selected patient outcomes were assessed using multilevel regression analysis. Five models were constructed - one for each dependent variable: A (direct nurse care), A1 (technical), A2 (information), A3 (interpersonal), B (indirect nursing care). Patient and nurse characteristics were included as control variables. These included age of nurse and patient, patient gender, nurse education, nurse experience (total and in unit) and patient days of hospitalization. Due to the design of the study it was impossible to link individual nurse rationing data to individual patient satisfaction scores, therefore unit-level rationing and RPPE measurements, as well as nurse characteristics measurements were used to define the significance of effects.

In order to compare the findings of the current study with past literature on the thresholds at which rationing began to affect these outcomes negatively, BERNCA scores were recorded into 6 levels: 0, 0.5, 1.0, 1.5, and 2.0, more than 2.5. The levels were based on relevant past literature (please see Schubert et al. [10,4] for a similar approach).

The level of significance was set at p < 0.05 and data were analysed using SPSS 19.0 for Windows (SPSS Inc. Chigaco, IL, USA).

## Results

### Demographics

Three hundred and fifty two (352) patients participated in the study and 33.8% (n = 119) were females. The age of all the patients ranged from 18 to 94 years, with a mean of 60.3 years (SD = 18.3). The length of their stay in the hospital ranged from 2 to 75 days, with a mean of 7.8 days.

For the nurse participants, seven hundred fifteen questionnaires (715) were distributed and four hundred and thirty three (433) were returned, a response rate of 60.6%. Three hundred ninety-three questionnaires (393) were considered eligible for analysis. The majority of the nurse participants were females, 70.1% (n = 278); the age range of the group was between 21 to 59 years, with a mean of 34.1 (SD = 9.4). Their total work experience ranged from 1 month to 40 years, with a mean of 11.4 years, while their experience at their department during data collection ranged from 1 month to 38 years, with a mean of 5.4 years. All the details regarding patients’ and nurses’ characteristics appear in Table 1.

**Table 1:** Nurse and Patients’ characteristics

	**Nurses (n = 393)**		**Patients (n = 352)**	
**Demographic variables**	**N (%)**	**Mean (SD)**	**N (%)**	**Mean (SD)**
Genderª Females	278 (70.1)		119 (33.8)	
Males	115 (29.0)		227 (64.5)	
Age		34.1 (9.4)		60.3 (18.3)
Experience in nursing (years)		11.4 (9.3)		
Experience in current department (years)		5.3 (5.5)		
Departmentª Surgical	211 (53.7)^b^		211 (59.9)^b^	
Medical	156 (39.7)^b^		137 (38.9)^b^	

ªThe total number varies due to missing data; ^b^Number of participants in each department.

### Aim 1: relationship between patient satisfaction with rationing of nursing care and work environment adjusting for patient and nurse characteristics

Before presenting the findings from the regression models, descriptive statistics were calculated for patient satisfaction, nurse-reported rationing and assessment of working environment. The mean level of all patient satisfaction factors was close to 4, indicating that on average patients reported to be very satisfied with the nursing care they received (see Table 2).

**Table 2:** Patient satisfaction level (range: 1–5)ª

**Patient satisfaction factors**	**Score range**	**Mean**	**SD**
A: Direct nursing Care	2.29-5.00	4.01	.64
A1: Technical Care subscale	2.25-5.00	4.17	.63
A2: Information subscale	1.00-5.00	3.96	.79
A3: Interpersonal relations subscale	2.00-5.00	3.92	.76
B: Indirect nursing care (food, cleaning, noise)	1.87- 5.00	4.03	.62

ªPatient satisfaction level: 1 = completely dissatisfied - 5 = completely satisfied.

The patient sub-scales were significantly inter-correlated. In particular, the direct nursing care factor (factor A: referring to the technical, information and interpersonal relations aspects of direct nursing care) was positively related to the indirect nursing care factor (factor B: referring to rest, cleanliness and food) (r = 0.59, p < 0.001), indicating that patients who were satisfied with aspects of nursing care were also satisfied with indirect nursing related care aspects. In addition, the three subscales of the nursing care factor were inter-related suggesting that patients who were satisfied with technical issues of their care (factor A1) were also satisfied with the information provided (factor A2) (r = 0.68, p < 0.001), interpersonal relations (factor A3) (r = 0.69, p < 0.001); similarly, patients satisfied with the information received (factor A2) were also satisfied with the level of interpersonal relations (factor A3) (r = 0.61, p < 0.001).

At the individual level, the mean score of implicit rationing of nursing care was 0.83 (SD = 0.52, range = 0–3) indicating that when asked how often they were unable to perform specific tasks, nurses reported this occurred almost rarely. The overall mean of RPPE was 2.76 (SD = 0.32, range = 1.28 – 3.69) suggesting that on average nurses tend to agree they have a satisfactory quality in their professional practice environment. In addition, the Kendall’s tau correlation coefficient between the two scales showed a small but significant correlation (τ = −0.234, p < 0.001), indicating that nurses who were not satisfied with their work environment (low level on RPPE) also reported that they frequently were unable to perform basic nursing tasks (high level on BERNCA).

For analytical purposes and to gain a wider view of the level of rationing of nursing care and quality of the nurse practice environment, departmental and hospital level mean scores were also calculated for each of the two scales and are presented in Table 3.

**Table 3:** Level of rationing and overall professional practice environment at departmental and hospital level

	**Medical**	**Surgical**	**H- 1ª**	**H - 2ª**	**H - 3ª**	**H - 4ª**	**H - 5ª**
**BERNCA**							
Mean	0.89	0.77	0.73	1.11	0.64	0.89	1.01
N	159	205	225	65	22	42	29
Standard Deviation	0.53	0.50	0.49	0.63	0.38	0.47	0.42
Minimum	0.50	0.00	0.00	0.00	0.00	11	0.25
Maximum	2.63	3.00	2.63	3.00	1.75	2.60	2.10
**RPPE**							
Mean	2.69	2.81	2.76	2.66	2.94	2.73	2.77
N	159	208	227	65	23	42	29
Standard Deviation	.28	0.32	0.28	0.39	0.38	0.31	0.26
Minimum	1.62	1.28	1.66	1.28	1.92	2.13	2.15
Maximum	3.49	3.69	3.59	3.69	3.59	3.41	3.28

ªH : Hospital 1, Hospital 2, Hospital 3, Hospital 4, Hospital 5.

Overall, there were significant differences in the levels of rationing amongst hospitals (0.64-1.10, p < 0.001) and departments (0.77-0.89, p = 0.025). Similarly, there were significant differences in the measured levels of RPPE amongst hospitals (2.66-2.94, p = 0.007) and departments (2.69-2.81, p < 0.001).

The main control characteristics that were significantly related to patient satisfaction were patient gender (where the results showed that women were on average more satisfied compared to men), total experience of nurses (higher experience was related to higher patient satisfaction) and number of days of hospitalization (more days were associated with higher patient satisfaction). Note that “nurse age” and “nurse experience” variables were included interchangeably in the models for multicollinearity purposes. Table 4 below shows the regression results both when the models were adjusted for the control variables (adjusted model) and not adjusted (unadjusted model), as well as when BERNCA and RPPE were included as single predictors or together.

**Table 4:** Regression results for the effect of rationing and/or professional environment on patient satisfaction

**Variables**	**Unadjusted model**				**Adjusted model**	
	**Beta (t)**	**p-value**	**R**^**2**^	**Beta (t)**	**p-value**	**R**^**2**^
**1. Dependent:**						
**Factor A-Direct nursing Care**						
Rationing	−1.11 (7.15)	<0.001^b^	0.13	−1.04 (−6.34)	<0.001^b^	0.14
RPPE	1.09 (4.01)	<0.001^b^	0.05	0.93 (2.94)	0.004^b^	0.06
Rationing &	−1.59 (−6.30)	<0.001^b^	0.14	−1.82 (−6.62)	<0.001^b^	0.17
RPPE	1.01 (2.41)	0.017		1.73 (3.47)	0.001^b^	
**2. Dependent:**						
**Factor A1-Technical Care**						
Rationing	−0.83 (−5.2)	<0.001^b^	0.07	−0.79 (−4.70)	<0.001^b^	0.08
RPPE	0.50 (1.83)	0.068	0.01	0.39 (1.25)	0.211	0.03
Rationing &	−1.59 (−6.25)	<0.001^b^	0.11	−2.17 (−4.29)	<0.001^b^	0.13
RPPE	1.59 (3.77)	<0.001^b^		1.74 (6.31)	<0.001^b^	
**3. Dependent:**						
**Factor A2-Information**						
Rationing	−0.89 (−4.46)	<0.001^b^	0.05	−0.78 (−3.69)	<0.001^b^	0.06
RPPE	1.11 (3.30)	0.001^b^	0.03	0.87 (2.21)	0.028ª	0.04
Rationing &	−0.97 (−2.97)	0.003^b^	0.05	−1.14 (−3.17)	0.002^b^	0.07
RPPE	0.17 (0.32)	0.750		0.80 (1.22)	0.224	
**4. Dependent:**						
**Factor A3-Interpersonal**						
Rationing	−1.60 (−9.13)	<0.001^b^	0.20	−1.55 (−8.42)	<0.001^b^	0.21
RPPE	1.68 (5.37)	<0.001^b^	0.08	1.55 (4.26)	<0.001^b^	0.09
Rationing &	−2.16 (7.57)	<0.001^b^	0.21	−2.48 (8.15)	<0.001^b^	0.25
RPPE	1.18 (2.47)	0.014ª		2.12 (2.79)	<0.001^b^	
**5. Dependent:**						
**Factor B-Indirect nursing Care**						
Rationing	−0.31 (−1.95)	0.052	0.01	−0.52 (3.16)	0.002^b^	0.08
RPPE	0.32 (1.21)	0.228	0.00	0.17 (0.56)	0.575	0.05
Rationing &	−1.24 (−4.91)	<0.001^b^	0.07	−1.26 (−4.62)	<0.001^b^	0.11
RPPE	1.97 (4.65)	<0.001^b^		1.69 (3.37)	0.001^b^	

^ª^Variable is significant at 0.05 level^b^Variable is significant at 0.01 level.

The main results from the regression table show that rationing and work environment were, in general, related to the five variables of patient satisfaction (factor A, factors A1-A3 and factor B) even after controlling for nurse and patient characteristics but with some exceptions. More specifically, rationing was consistently related to patient satisfaction both alone and after controlling for patient and nurse characteristics or the work environment metric. The only exception was its relationship with indirect nursing care (factor B) where that relationship, when it was included alone, was marginally significant (p = 0.052), but became significant after adjusting for the control variables and work environment. Overall, higher levels of rationing were significantly related to lower levels of patient satisfaction.

Similarly the work environment was related to patient satisfaction, both alone and after controlling for patient and nurse characteristics, for all patient satisfaction outcomes except technical care (factor A1) and indirect nursing care (factor B). When the association was significant, higher scores on the RPPE scale were associated with higher patient satisfaction.

However, it should be noted that for both of the above patient satisfaction factors i.e. technical care (factor A1) and indirect nursing care factor (factor B), the effect of work environment became significant when rationing was also included in the model, showing that the two predictors had a combined interactive effect on the two patient satisfaction outcomes. Then again, the relationship of the work environment with information (factor A2) became non-significant when rationing was included in the model, indicating the significant role of rationing in patient satisfaction regarding information over work environment when both predictors are entered together in the model.

### Aim 2: identifying threshold levels of rationing in relation to patient satisfaction

When considering the unit-level aggregate nurse metric of BERNCA, we had levels of only 0.5 and 1 of rationing. The results from the adjusted regression models (see Table 5) showed that even at the lowest level of rationing i.e. 0.5 patients indicated low satisfaction for both direct nursing care (factor A), indirect nursing care (factor B) as well as for technical care, information, and interpersonal relations (subscales of direct nursing care: factors A1-A3 respectively). The trend of a negative association between rationing and patient satisfaction factors was significant for direct nursing care (factor A), technical care (factor A1) and interpersonal relations (factor A3). For higher levels of rationing i.e. 1, the pattern of negative association between rationing and satisfaction factors continued, although they were insignificant.

**Table 5:** Rationing threshold levels for patient satisfaction

**Patient satisfaction factors**	**Rationing level: 0.5 (N = 272)**			**Rationing level: 1 (N = 58)**		
	**Beta (t)**	**p-value**	**R**^**2**^	**Beta (t)**	**p-value**	**R**^**2**^
Factor A-Direct nursing Care	−1.676 (−2.77)	0.006^b^	0.04	−0.438 (−1.09)	0.281	0.15
Subscale A1-Technical Care	−1.208 (−1.96)	0.05ª	0.04	−0.387 (−1.07)	0.290	0.25
Subscale A2 -Information	−0.857 (−1.09)	0.276	0.01	−0.901 (−1.39)	0.169	0.12
Subscale A3-Interpersonal relations	−2.880 (−4.19)	<0.001^b^	0.08	−0.025 (−0.07)	0.942	0.08
Factor B-Indirect nursing Care	−0.941 (−1.56)	0.121	0.11	−0.182 (−0.49)	0.626	0.14

ªVariable is significant at 0.05 level, ^b^Variable is significant at 0.01 level ^b.^

## Discussion

The main finding of our study is the negative association of the two main variables i.e. reports of the rationing of nursing care and perceived professional practice environment as viewed with respect to patient satisfaction concerning hospital care, after adjusting for patient and nurse characteristics. These results support the relationship suggested between organizational and environmental variables, plus care rationing and patient outcomes as described in the theoretical model of implicit rationing of nursing [6]. Although the average rationing levels were not high, in line with similar studies [4,12] the related analyses provided estimates of the effect of implicit rationing of nursing care and nurses’ perceptions of their professional practice environment after controlling for patient and nurse covariates, confirming previous findings [6,51].

To our knowledge this is the first study that examines patient satisfaction as an outcome of nursing care rationing using a multidimensional satisfaction measuring instrument. Although there is evidence that higher levels of implicit rationing of nursing care resulted in significant decrease in the probability of patient satisfaction [4,51], the present study gives a further explanation of the different aspects of patient satisfaction and how they are related to environmental variables and rationing of nursing care. For example, indirect nursing care including food, cleanliness and minimization of noise as well as the information subscale, were not related to patient satisfaction, compared with aspects of direct care including interpersonal relationships and technical care that were found to be strongly and significantly related with care rationing.

There is evidence that patients are particularly satisfied from the technical aspect of care [16,24,52] and that interpersonal factors contribute strongly to patient satisfaction [53,54]. A concept analysis of patient satisfaction from nursing care describes affective support and technical competencies as the attributes leading to the health outcome of patient satisfaction with nursing care [14]. Although there is some debate as to whether patients are able to judge the technical aspects of care [55], most of the literature supports that patients’ judgments of interpersonal characteristics are the strongest predictors of satisfaction [56,57]. A study that evaluated patient satisfaction with both qualitative and quantitative approach found that patients gave the highest ratings to technical care, but the qualitative analysis revealed that the interpersonal aspect of care was central to patients’ experience. Berg et al. [57] in examining the effect of technical care and interpersonal care on general care, found strong relationships between the three variables and an important effect of interpersonal care on technical care meaning that if patients rated nurses as sensitive and sympathetic, they also rated them as competent, educated and experienced.

The negative relationships of care rationing and satisfaction from the interpersonal and technical aspects of care found in this study, means that we need to further explore and understand the associations of care rationing within the complexities of carer/patient relationships.

The study also aimed to identify the threshold at which rationing of nursing care is significantly associated with negative patient outcomes, specifically patient satisfaction. The statistical analysis indicated that the lowest level of rationing (0.5) was significantly related to patient satisfaction from direct nursing care including technical care and interpersonal relations. Although eliminating nursing care rationing might be considered impossible within the current economic and organizational constraints, this finding gives an indication as to the point at which rationing begins to affect patient outcomes and could become a serious threat to patient safety. The BERNCA instrument used in our study provided a clinically meaningful method for tracking the effects of low resources [4] on patient satisfaction as related to nursing care and can be used to investigate also other care related outcomes.

Also, the way that nurses perceive their professional environment had a significant effect on patient satisfaction. Although the different measuring tools used in the related studies do not allow for safe comparisons, the findings of this study go some way to confirming this relationship [19,27].

Rationing of nursing care and the practice environment are highly correlated suggesting that nurses may decide to ration, omit or delay care according to the perceptions they have towards the environment of care delivery (low levels on RPPE were related with high levels on the BERNCA). This is confirmed also by the finding that in hospitals with high nurses’ scores of professional practice environment, the level of rationing is low when compared to the hospitals in which the professional environment is not so favourably rated by nurses.

This study has several strengths and a number of weaknesses, therefore when drawing generalizations caution needs to be exercised. Although the sample size was justified by the requirements of statistical power analysis, the study was conducted in a small country in which the health system suffers from a number of inefficiencies and is currently in a state of transition [58]. On the other hand, although generalisability of the results is limited within the country, the fact that the sample was drawn from all the general state hospitals strengthens the findings of rationing and its correlations to patient satisfaction and nurses’ perceptions of their practice environment in the particular country.

A further weakness is the number of factors that may intervene in the data collection process to cause random error. These include variations in the administration of the questionnaires in the different units as well as the lengthy period of data collection (1 year) that may have resulted in possible fluctuations of patient satisfaction levels. The relatively high percentage of the non-respondent nurses may also indicate the sensitivity of the subject area and the possible reluctance of nurses to admit omissions in their work.

## Conclusions

In this study, rationing of nursing care appears as an organisational difficulty, associated with the way nurses perceive environmental constraints of practicing their profession and it is linked with patient outcomes such as patient satisfaction from nursing care. The findings have several implications for nursing practice, management and research. Firstly, nursing care rationing needs to be openly recognized as a problematic area in nursing and a threat to patient safety [7] that requires consideration in policy development. The identification of thresholds at which rationing starts to influence patient outcomes in a negative way may allow nurse managers to monitor rationing levels and react accordingly. Conceptualising rationing and developing interventions that improve nurse-patient interaction, relationships and improve outcomes such as patient satisfaction is also crucial at a clinical and managerial level.

Secondly, there is a need to understand several unexplored aspects in the multifaceted area of caring, such as the factors influencing care rationing, nurses’ critical thinking and decision-making processes, and the criteria used by nurses to allocate and distribute their resources among patients.

## Competing interests

The authors declare that they have no competing interests.

## Authors’ contributions

EP designed the study, supervised the data collection and wrote the paper. PA assisted in the supervision of the data collection and with the writing of the article. HT carried out the statistical analysis and assisted with the writing of the article. AM assisted writing and finalising the manuscript. All authors read and approved the final manuscript.

## Pre-publication history

The pre-publication history for this paper can be accessed here:

http://www.biomedcentral.com/1472-6955/13/26/prepub
